# Injection Drug User Quality of Life Scale (IDUQOL): Findings from a content validation study

**DOI:** 10.1186/1477-7525-5-46

**Published:** 2007-07-30

**Authors:** Anita M Hubley, Anita Palepu

**Affiliations:** 1 Measurement, Evaluation, and Research Methodology, Department of Educational and Counselling Psychology, and Special Education, University of British Columbia, Vancouver, BC, Canada; 2 Division of Internal Medicine, Department of Medicine, University of British Columbia, Vancouver, BC, Canada; 3 Department of Medicine, St. Paul's Hospital, Vancouver, BC, Canada

## Abstract

**Background:**

Quality of life studies among injection drug users have primarily focused on health-related measures. The chaotic life-style of many injection drug users (IDUs), however, extends far beyond their health, and impacts upon social relationships, employment opportunities, housing, and day to day survival. Most current quality of life instruments do not capture the realities of people living with addictions. The Injection Drug Users' Quality of Life Scale (IDUQOL) was developed to reflect the life areas of relevance to IDUs. The present study examined the content validity of the IDUQOL using judgmental methods based on subject matter experts' (SMEs) ratings of various elements of this measure (e.g., appropriateness of life areas or items, names and descriptions of life areas, instructions for administration and scoring).

**Methods:**

Six SMEs were provided with a copy of the IDUQOL and its administration and scoring manual and a detailed content validation questionnaire. Two commonly used judgmental measures of inter-rater agreement, the Content Validity Index (CVI) and the Average Deviation Mean Index (AD_M_), were used to evaluate SMEs' agreement on ratings of IDUQOL elements.

**Results:**

A total of 75 elements of the IDUQOL were examined. The CVI results showed that all elements were endorsed by the required number of SMEs or more. The AD_M _results showed that acceptable agreement (i.e., practical significance) was obtained for all elements but statistically significant agreement was missed for nine elements. For these elements, SMEs' feedback was examined for ways to improve the elements. Open-ended feedback also provided suggestions for other revisions to the IDUQOL.

**Conclusion:**

The results of the study provided strong evidence in support of the content validity of the IDUQOL and direction for the revision of some IDUQOL elements.

## Background

In the health and medical fields, quality of life (QoL) is widely used to evaluate social and clinical interventions, treatment side effects, and disease impact over time [1,2]. Most of these QoL instruments tend to focus on health-related quality of life (HRQOL) or the functional effects of respondents' perceived mental and physical health [3]. Gill and Feinstein [3], however, defined QoL as a reflection of respondents' perceptions and reactions to not only their mental and physical health, but also to non-health related aspects of their lives (e.g., family, friends, work). Thus, measurement of QoL needs to encompass more than just the health-related aspects of respondents' lives.

Nearly all studies of QoL in injection drugs users (IDUs) use measures of HRQOL [4-8]. Commonly used measures of HRQOL with this population include the Opiate Treatment Index [9], Nottingham Health Profile [10,11], the Berlin Quality of Life Profile [12] and the MOS surveys (including the SF-36 and the SF-12) [13-16]. Many studies have shown that IDUs experience significantly lower HRQOL relative to the general population [16-20] but, as noted by Fernández Miranda [21], remarkably little published research has examined QoL as an outcome variable in the treatment of drug addiction [22,23].

Although previous research with IDUs and related populations (e.g., illicit drug users, HIV/AIDS) has considered the effects of non-health related aspects of respondents' lives on their HRQOL [4,7,15,24-28] or even on the initiation or maintenance of drug use [29-35], rarely has published research with IDUs used a broadly-defined QoL measure (i.e., one that captures various social, psychological, physical, geographic, and occupational domains of QoL). Two exceptions would be Wasserman and colleagues [36], who examined the psychometric properties of Lehman's [37] Quality of Life Interview – Brief Version with IDUs, and Dunaj and Kovác [38], who compared convicted drug addicts and controls on broadly-defined QoL using the WHOQOL-BREF [39] and ComQol-A5 [40]. Dunaj and Kovác reported that addicts scored significantly lower than controls in their subjective ratings of areas such as health, emotional well-being, safety, and social standing.

Although broader measures of QoL are beginning to be used with IDUs [36,38] and are certainly an improvement over the use of strictly HRQOL measures, measures developed specifically for the IDU population and using a context sensitive approach that considers the many life areas deemed by IDUs as critical to their QoL, are still needed. QoL, as defined by the World Health Organization Quality of Life (WHO-QOL) group, refers to "an individual's perceptions of their position in life in the context of the culture and value systems in which they live, and in relation to their goals, expectations, standards, and concerns" (pp. 1–2) [41]. The item content and methods of administration for most available QoL instruments do not measure the QoL of drug users in a culturally-sensitive fashion [42]. IDUs live in a distinct environment characterized by a high prevalence of infectious disease, crime, violence, and lack of stable housing. Many IDUs cannot depend on basic necessities and experience considerable instability in many aspects of their lives.

A recently developed broadly-defined QoL measure, the Injection Drug User Quality of Life (IDUQOL) scale, was designed to capture the health and non-health related aspects of IDUs' lives that would be important components of their quality of life, particularly given their individual circumstances and environment [43,44]. This measure has also been adapted for use in Spanish with injection and non-injection drug users [45]. To use an instrument with confidence, it is important that there be evidence of validity – that is, the meaningfulness, usefulness, and appropriateness of an instrument for a given population in a given context [46-48]. Previous research has examined the factor structure, internal consistency, and test-retest reliability of scores from the IDUQOL as well as the criterion-related, convergent, and discriminant validity of inferences made from the measure [44]. Content validity, a critical step in the test development and validation process [49-51], refers to the degree to which elements of an assessment tool are representative of the construct of interest and appropriate for a given population [52]. Importantly, the elements of interest in a content validation study are not just the content or items of the measure, but all elements of the instrument including the instructions, response format, and scoring procedures [53].

The purpose of the present study was to examine the content validity of the IDUQOL using judgmental methods based on subject matter experts' (SMEs) ratings of the IDUQOL title, items, instructions, response format, scoring procedures, and record form.

## Methods

### Participants

The sample consisted of a panel of six subject matter experts (SMEs; 50% male), all of whom were researchers working in the area of drug use in the United States or Canada with an average of 10 years experience in the field. Two SMEs were epidemiologists and four SMEs were physicians who also provided addiction and medical care to drug users in their clinical practice. As noted by others [54], there is no set number of SMEs required for content validation studies. Typically, somewhere between three to ten experts is recommended, although a minimum of five SMEs is recommended to control for chance agreement; furthermore, the larger the number of experts, the greater the confidence in the ratings and the easier it is to detect rater outliers [53,55].

### Measures

The subject matter experts were provided with a copy of the IDUQOL and its administration and scoring manual and a detailed content validation questionnaire. Ethics approval for this study was obtained from the University of British Columbia and Providence Health Care Research Ethics Boards.

#### Injection Drug User Quality of Life Scale (IDUQOL)

The original IDUQOL, which includes both health and non-health related aspects of QoL [3] and is based on the WHO-QOL group definition of QoL [41], consisted of 20 life areas. Several of these areas (e.g., Drugs, Drug Treatment, Harm Reduction and Neighbourhood Safety) were included in the measure precisely because of their particular relevance to the social and physical reality of IDUs as confirmed by focus groups during the development phase [43]. Each IDUQOL life area is represented on a 4 by 4 inch card, with the name of the area printed on the front along with a simple picture. A description of the life area is presented on the back (see Table 1 for a list of all 20 life areas and descriptions). Graphic representation of the life areas is intended to make the instrument more accessible to individuals who have low literacy skills or do not speak English as a first language. When administering the IDUQOL, the interviewer starts by showing the respondent each of the 20 life area cards and describes the area. The participant selects those areas that he/she deems important to his/her quality of life and any remaining cards are set aside. The cards representing important areas are laid out and the participant is given three poker chips for each card. The total number of chips can, therefore, range from 0 (no life areas are important) to 60 (all 20 life areas are important). The participant then distributes the chips across the cards to indicate the level of importance of each life area, with more chips indicating greater importance. Next, the participant provides a satisfaction rating for each area, using a 6-point Likert-type scale anchored by 1 (very dissatisfied) and 6 (very satisfied) and illustrated with six stylised frowning and smiling faces.

**Table 1:** Injection Drug User Quality of Life (IDUQOL) life area names and descriptions

Life Area	Description
Being Useful	e.g., volunteering, employment, participating in the community, helping others
Community Resources	e.g., food bank, soup kitchen, shelters, outreach programs, social service agencies
Drugs	drug use – e.g., alcohol, heroin, cocaine, crack
Drug Treatment	e.g., detox, recovery house, residential treatment, methadone, abstinence
Education	e.g., formal schooling, literacy programs
Family	e.g., parents, children, siblings, foster families (not friends)
Feeling Good about Yourself	e.g., self-esteem, self-worth
Friends	anyone you consider a friend (but not family)
Harm Reduction	access to, and experience with: e.g., methadone treatment, needle exchange, safe injection programs, prescription heroin
Health	mental and physical health, including HIV, AIDS, Hepatitis C, disability, schizophrenia
Health Care	access to, and experience with: physicians, nurses, hospitals, clinics, ER
Housing	e.g., owning, renting, house, apartment, hotel room, shelters, homeless
Independence and Free Choice	e.g., making your own decisions, autonomy, being able to do things on your own, having individual rights
Leisure Activities	e.g., music, sports, movies, books, parties
Money	e.g., income, welfare, cash flow, meeting your needs
Neighborhood Safety	e.g., crime, violence, police harassment
Partner(s)	e.g., spouse, common-law partner, same-sex partner, girlfriend or boyfriend (not casual partners)
Sex	e.g., sexual intimacy, quantity or quality of sex, sex in exchange for money or drugs, sexual abuse
Spirituality	e.g., religion, faith, belief in a higher being or spiritual world (or not)
Transportation	e.g., car, taxi, public transportation, getting to places you need to go

When scoring the IDUQOL, the importance rating (number of chips) of each area is divided by the total number of chips used by that participant and then multiplied by the satisfaction rating for that area. This produces an area score. Finally, all area scores are summed to obtain an overall quality of life score ranging from 1 (very dissatisfied) to 6 (very satisfied).

#### IDUQOL content validation questionnaire

The questionnaire was divided into seven sections covering the clarity of the instrument title, ease of administration procedure instructions, clarity of the names and descriptions of the 20 IDUQOL life areas, whether each of the 20 IDUQOL life areas should be included in the measure (including whether any life areas need to be added, revised, or deleted), ease of the response formats used for each of importance and satisfaction ratings, clarity of scoring procedure instructions, and the ease of use of the record form. Experts were also given the opportunity to provide open-ended commentary in each section. As recommended by Lynn [55], a four-point Likert type scale was used in most cases. For questions involving clarity, the following four response options were used: 0 = not at all clear, 1 = somewhat clear, 2 = mostly clear, 3 = very clear. For questions involving ease, the following four response options were used: 0 = not at all easy to follow/use, 1 = somewhat easy to follow/use, 2 = mostly easy to follow/use, 3 = very easy to follow/use. For questions involving inclusion of items, the following three response options were used: 0 = no, 1 = unsure, 2 = yes. Two questions asked about how helpful the provided examples were in the manual; for these, the following four response options were used: 0 = not at all helpful, 1 = somewhat helpful, 2 = mostly helpful, 3 = very helpful.

### Procedures

The six SMEs were identified through the second author's professional contacts with nationally and internationally recognized experts in the area of substance abuse epidemiology and treatment. They were sent a letter of invitation and agreed to take part in the study. None of the SMEs were associated with the development of the IDUQOL. The SMEs were mailed a copy of the IDUQOL (which included the 20 life area cards, poker chips, satisfaction rating card, and record form), the administration and scoring manual, and the IDUQOL content validation questionnaire. As suggested by Grant and Davis [56], SMEs were provided with the conceptual basis for the IDUQOL via the brief introduction in the manual in which the definition of QoL underlying this measure, the target population, and how the measure is intended to be used was provided. The SMEs completed the content validation questionnaire at their leisure and independently of one another. All SMEs returned usable questionnaires.

Two commonly used judgmental measures of inter-rater agreement, the Content Validity Index (CVI) [55,57] and the Average Deviation Mean Index (AD_M_) [58-60], were used to evaluate SMEs' agreement on ratings of the various IDUQOL elements. The two measures provide very different types of information, however, and should be viewed as complementary. Generally, the CVI indicates the proportion of SMEs that endorse an element as content valid whereas the AD_M _indicates the degree of disagreement among SMEs in the response option selected regardless of whether they, as a group, endorsed an element or not. Thus, one should first examine the CVI values to determine whether the SMEs endorsed an item or not and then consider the level of agreement among the SMEs by examining the AD_M_.

The CVI can be computed at the individual item level (I-CVI) and at the level of the overall scale or subscale (S-CVI). I-CVI is computed as the proportion of SMEs that endorse an item. Following standard procedures for four response options [49,55], ratings of 2 or 3 were combined and treated as endorsements by SMEs whereas ratings of 0 or 1 were combined and treated as non-endorsements in the present study. When three response options were used, a rating of 2 was treated as an endorsement by SMEs whereas ratings of 0 or 1 were combined and treated as non-endorsements. A minimum of five out of the six SMEs (I-CVI ≥ .83) had to endorse an item to achieve significant evidence (α = .05) of content validity for any given item on the IDUQOL content validity questionnaire and to provide confidence that agreement was not occurring by chance alone [55]. Elements that were not endorsed by a minimum of five SMEs were examined further to determine if appropriate revisions could be made.

The S-CVI may be defined and computed a number of different ways, but Polit and Beck [49] recommend using the average proportion of items endorsed by the SMEs (what they refer to as S-CVI/Ave) and computing this as the average of the I-CVI values. This is the approach that will be used in the present study in conjunction with Lynn's [55] description of S-CVI as "the proportion of total items judged content valid" (p. 384). For S-CVI/Ave, the minimum acceptable value is recommended to be .90 [49].

The AD_M _Index measures dispersion of ratings about the mean rating; thus, it is actually a measure of disagreement so lower values indicate higher levels of agreement among SMEs. An advantage of the AD_M _Index is that it provides a measure of dispersion that is directly interpretable in terms of the original rating scale units. The general cut-off for determining acceptable AD_M _values is based on c/6, with c referring to the number of response options [59]. Thus, using this guideline for practical significance, acceptable AD_M _values are .50 or less for ratings with three response options and .69 or less for ratings with four response options. Critical values that can be used to evaluate whether an obtained AD_M _could have been achieved by chance can also be computed. Critical values for AD_M _at the 5% level of significance, taking into account the number of SMEs and the number of response options in the present study, would be .28 or less for three response options and .44 or less for four response options [58]. AD_M _values that are equal to or below these critical values are unlikely to have been obtained by chance. Elements for which AD_M _values were neither practically nor statistically significant or were only practically significant were examined further to determine if appropriate revisions could be made.

## Results

### Content validity evidence for IDUQOL life areas

Table 2 presents the I-CVI and AD_M _results for each of the 20 IDUQOL life areas indicating whether (a) each life area was appropriate for a QoL measure for IDUs, (b) the name of the life area was clear, and (c) the description of the life area was clear. I-CVI results showed that all individual life areas, including the name used and the description provided, were endorsed by a minimum of five SMEs.

**Table 2:** Item level CVI (I-CVI) and AD_M _index values for each of the 20 IDUQOL life areas

	Appropriate?^a^		Name^b^		Description^b^	
Life Area	I-CVI	AD_M_	I-CVI	AD_M_	I-CVI	AD_M_
Being Useful	1.00	.00	1.00	.44	0.83	**.67**
Community Resources	1.00	.00	1.00	.28	1.00	.28
Drugs	1.00	.00	0.83	**.55**	0.83	**.67**
Drug Treatment	0.83	.28	1.00	.00	1.00	.28
Education	0.83	.28	1.00	.00	1.00	.28
Family	1.00	.00	1.00	.00	1.00	.44
Feeling Good about Yourself	0.83	.28	1.00	.28	1.00	**.50**
Friends	1.00	.00	1.00	.00	1.00	.28
Harm Reduction	1.00	.00	1.00	.00	0.83	.33
Health	1.00	.00	1.00	.00	1.00	.28
Health Care	1.00	.00	1.00	.00	1.00	.28
Housing	1.00	.00	1.00	.00	1.00	.00
Independence and Free Choice	0.83	.28	1.00	**.50**	1.00	.00
Leisure Activities	1.00	.00	1.00	.00	1.00	.00
Money	1.00	.00	1.00	.00	1.00	.28
Neighborhood Safety	1.00	.00	1.00	.00	1.00	.44
Partner(s)	1.00	.00	1.00	.28	1.00	.00
Sex	1.00	.00	1.00	.00	1.00	.44
Spirituality	1.00	.00	1.00	.00	1.00	.00
Transportation	1.00	.00	1.00	.00	1.00	.00

^a ^Ratings were made on a 3-point scale. ^b ^Ratings were made on a 4-point scale.

Note. I-CVI: 1.00 = endorsement by all six subject matter experts (SMEs); 0.83 = endorsement by five of six SMEs. AD_M _Index: with a 3-point scale, acceptable values are .50 or less and statistically significant values are .28 or less; with a 4-point scale, acceptable values are .69 or less and statistically significant values are .44 or less. Values that are acceptable, but not statistically significant, are bolded.

The AD_M _results provide additional information about the extent to which the SMEs agreed on the exact rating (e.g., not at all clear, somewhat clear, mostly clear, very clear) for each life area. In all cases, acceptable agreement (i.e., practical significance) was obtained. In terms of the appropriateness of the life areas, each life area also showed statistically significant agreement among the SMEs. When considering whether the *name *of the life area was clear, there were only two cases (i.e., Drugs, Independence & Free Choice) in which agreement was acceptable, but not statistically significant – meaning that agreement could have occurred by chance. In the case of Drugs, five SMEs rated this name as 'very clear' whereas one SME thought it was only 'somewhat clear' because drugs could be confused with medications. Independence & Free Choice was rated as 'mostly clear' by half of the SMEs and 'very clear' by the other half of SMEs with the problem being different interpretations of the word "independence". When considering whether the *description *of the life area was clear, there were three cases (i.e., Being Useful, Drugs, Feeling Good about Yourself) in which agreement was acceptable, but not statistically significant. The description for Being Useful was rated as 'somewhat clear' by one SME, 'mostly clear' by one SME and 'very clear' by four SMEs. Most SME comments were focused on the visual depiction provided on the card and no suggestions for changes or additions to the description were offered. The description for Drugs (i.e., "drug use – e.g., alcohol, heroin, cocaine, crack") was rated as 'somewhat clear' by one SME, 'mostly clear' by two SMEs and 'very clear' by three SMEs. There were two concerns raised by SMEs. The first and most prominent concern was that we limited this life area to *use *of drugs; the second concern was that we only listed four drugs. The description for Feeling Good about Yourself (i.e., "e.g., self-esteem, self-worth") was rated as 'mostly clear' by half of the SMEs and 'very clear' by the other half of the SMEs. No suggestions were made for changes to the description.

SMEs were also asked if there were any life areas that they would recommend deleting or adding to the IDUQOL. No life areas were recommended for deletion from the IDUQOL. The following additional life areas were suggested: food, pets, personal safety, sense of future (e.g., hopefulness, aspirations), employment (as its own life area separate from Being Useful), and pain.

Table 3 shows that the S-CVI/Ave for the element groupings of Appropriateness, Name clarity, and Description clarity of the IDUQOL life areas ranged from .97 to .99, which exceeded the minimum value of .90 and is also strong evidence of content validity.

**Table 3:** Scale level CVI (S-CVI/Ave) for elements of the IDUQOL measure and manual

IDUQOL Element	S-CVI/Ave
Appropriateness of IDUQOL Life Areas (20 items)^a^	0.97
Clarity of IDUQOL Life Area Names (20 items)^b^	0.99
Clarity of IDUQOL Life Area Descriptions (20 items)^b^	0.98
Clarity of Title and Target Population (2 items)^b^	0.92
Ease of Administration Procedure (4 items)^b^	1.00
Ease of Response Formats (2 items)^b^	0.83
Ease of Scoring Procedure (3 items)^b^	0.94
Helpfulness of Provided Examples (3 items)^b^	1.00
Ease of the Record Form to Use (1 item)^b^	1.00

^a ^Ratings were made on a 3-point scale. ^b ^Ratings were made on a 4-point scale.

Note. Ease of Record Form result is the same as the individual level findings; this element is included here for completeness. For S-CVI/Ave, the minimum acceptable value is .90. A scale level AD_M _Index could not be applied here as it requires scales comprised of essentially parallel items.

### Content validity evidence for other IDUQOL elements

Table 3 also shows that the S-CVI/Ave for the element groupings of Clarity of Title and Target Population, Ease of Administration Procedure, Ease of Scoring Procedure, and Helpfulness of Provided Examples ranged from .92 to 1.00. Only Ease of Response Formats produced a S-CVI/Ave (.83) that was below the minimum recommended value of .90, although both items were endorsed by five out of six SMEs.

I-CVI and AD_M _results for other individual elements (e.g., title, administration instructions, scoring instructions, record form) of the IDUQOL measure and manual are presented in Table 4. I-CVI results showed that all individual elements were endorsed by a minimum of five SMEs. The AD_M _results show that acceptable agreement (i.e., practical significance) was obtained in all cases, although there were four cases in which agreement was not statistically significant (i.e., Clarity of Title, Response Format – Chips, Response Format – Smiley Faces, Scoring Procedure – Summed Score).

**Table 4:** Item level CVI (I-CVI) and AD_M _index values for IDUQOL measure and manual elements

IDUQOL Content Validity Questionnaire Item^a^	I-CVI	AD_M _Index
Clarity of Title	0.83	**.67**
Clarity of Intended Population	1.00	.00
Administration Procedure – Introduction	1.00	.28
Administration Procedure – Respondent Selects Life Areas	1.00	.44
Administration Procedure – Respondent Rates Importance	1.00	.00
Administration Procedure – Respondent Rates Satisfaction	1.00	.00
Response Format – Easy for Respondent to Use Chips	0.83	**.67**
Response Format – Easy for Respondent to Use Smiley Faces	0.83	**.67**
Scoring Procedure – Relative Importance Score	1.00	.44
Scoring Procedure – Importance × Satisfaction Score	1.00	.44
Scoring Procedure – Obtain Summed Score	0.83	**.67**
Example – Relative Importance Score	1.00	.00
Example – Importance × Satisfaction Score	1.00	.00
Example – Completed Sample Record Form	1.00	.00
Ease of the Record Form to Use	1.00	.28

^a ^Ratings were made on a 4-point scale.

Note. I-CVI: 1.00 = endorsement by all six subject matter experts (SMEs); 0.83 = endorsement by five of six SMEs. AD_M _Index: with a 4-point scale, acceptable values are .69 or less and statistically significant values are .44 or less. Values that are acceptable, but not statistically significant, are bolded.

The name or title of the IDUQOL was rated as 'somewhat clear' by one SME, 'mostly clear' by one SME and 'very clear' by four SMEs, with no suggestions made for how to make the title clearer. The Response Format – Chips was rated as 'somewhat easy to use' by one SME, 'mostly easy to use' by one SME, and 'very easy to use' by four SMEs. Suggestions were made for simplifying the instructions given to IDUs about how to use the poker chips to indicate the importance of the different life areas. In addition, it was suggested that poker chips might act as a trigger for IDUs with gambling issues. The Response Format – Smiley Faces was rated as 'somewhat easy to use' by one SME and 'very easy to use' by five SMEs. It was suggested that we consider using an odd-numbered Likert-type scale (rather than our 6-point Likert-type scale) for the smiley faces that would permit a neutral response. The Scoring Procedure – Summed Score was rated as 'somewhat easy to follow' by one SME, 'mostly easy to follow' by one SME, and 'very easy to follow' by four SMEs. It was suggested that we clarify the headings on the record form so they would better match the terms used in the manual.

### Suggested revisions to the IDUQOL based on SME feedback

Open-ended feedback and comments from the SMEs resulted in several other suggestions for changes to the IDUQOL measure, materials, and manual. These may be grouped into three points. First, suggestions were made to expand the descriptions for (a) Education, (b) Family, and (c) Sex. Second, it was pointed out that we needed to show greater diversity in our cards involving people – specifically Family and Friends. Third, we were advised to revise the cards depicting (a) Being Useful, and (b) Independence and Free Choice to make them clearer.

## Discussion

Test development and validation are ongoing processes designed to ensure measures and the inferences made from them remain appropriate, relevant, and useful for the target population and context of use [47]. The IDUQOL was developed as a measure of broadly defined subjective QoL that incorporates both health and non-health related aspects of IDUs' lives. Administration of the IDUQOL was designed to be sensitive to the diversity of literacy levels, English language skills, attention levels, and cognitive abilities of the target population. An important step in test development and validation is the evaluation of content validity. The purpose of the present study was to examine the content validity of various elements of the IDUQOL measure and manual using SMEs and two commonly used judgmental methods (i.e., CVI and AD_M_). The CVI indicates the proportion of SMEs that endorse an element as content valid whereas the AD_M _indicates the degree of disagreement among SMEs in the response option selected. Overall, the results of this study provide strong evidence for the content validity of the elements of the IDUQOL measure and manual. Specifically, the I-CVI results supported the content validity of each of the individual elements. These elements include the appropriateness, name, and description of each of the 20 life areas, clarity of the name of the measure, clarity of the target population, ease of each step of the administration procedure, ease of each response format (i.e., chips and smiley face scale), ease of each step of the scoring procedure, helpfulness of each of the provided example boxes in the manual, and ease of use of the IDUQOL record form.

The S-CVI/Ave results also supported the content validity of all of the grouped elements of the IDUQOL measure and manual (e.g., Appropriateness of Life Areas, Ease of Administration Procedure), with the exception of Ease of Response Formats. Two points are worth noting about the Ease of Response Formats case. First, when examined individually using I-CVI, each of the two items under Ease of Response Formats was endorsed by five of the six SMEs, supporting their content validity. In fact, the one SME who supposedly did not endorse either of these items actually circled both 'somewhat easy to use' and 'mostly easy to use' (responses that fell into the 'not endorsed' and 'endorsed' categories, respectively) in each case and indicated that the ease of each response format for the target population was an empirical question that should be piloted instead. Using a conservative approach, we treated this SME's response as a non-endorsement, although it could be argued to be more ambiguous. Second, it should be noted that when there are only two or three items making up a grouping of elements (as is the case for the two-item Ease of Response Formats grouping), the minimum acceptable level of .90 for S-CVI/Ave cannot be reached unless all but one item in the grouping achieves endorsement by all six of the SMEs. Taking each of these points into account, we would argue that the content validity of the response formats should not be discounted, but that care should be taken to ensure, through further study or pilot testing, that these response formats are appropriate to the group of IDUs with whom a researcher or practitioner wishes to use the IDUQOL. Based on interviews we conducted with the experienced staff who administered the IDUQOL to participants in another study [44,61], that sample of IDUs did not have difficulty using either response format, although some respondents expressed the desire for a neutral response option on the Likert-type smiley face satisfaction scale.

In terms of the AD_M _results, practical significance was obtained for all 75 elements of the IDUQOL and manual, indicating a high level of agreement among the SMEs in their ratings. However, statistical significance was not reached for the following nine elements: (a) names for the Drugs and Independence & Free Choice life areas, (b) descriptions for the Being Useful, Drugs, and Feeling Good about Yourself life areas, and (c) clarity of title, ease of response format – chips, ease of response format – smiley faces, and ease of scoring procedure – summed score. Thus, for these elements, it is possible that SME agreement on the ratings may have occurred by chance alone.

Both the CVI and AD_M _results and the open-ended feedback from the SMEs were used to make seven main revisions to IDUQOL elements. First, the names for the Drugs and Independence & Free Choice life areas were changed to Drugs & Alcohol and Free Choice, respectively. We also decided to take one SME's suggestion to change the Education life area to Education & Training.

**Figure 1:**
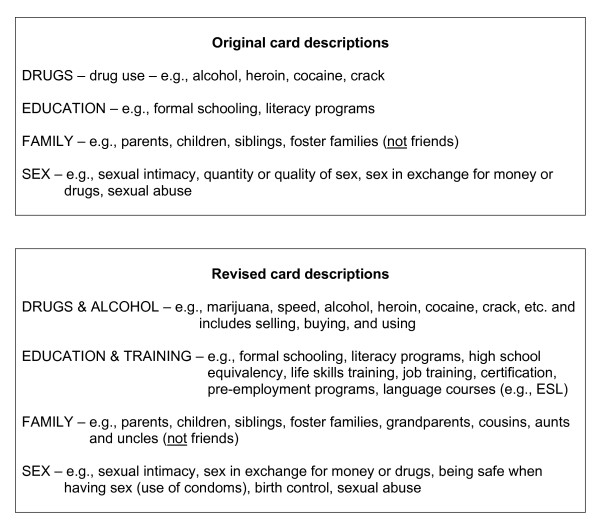
Samples of original and revised IDUQOL card descriptions.

Second, descriptions for the life areas of Drugs & Alcohol, Education, Family, and Sex were expanded. The original and revised descriptions for each of these life areas are provided in Figure 1. The descriptions for some other life areas (e.g., Being Useful, Feeling Good about Yourself) remained unchanged because SMEs had only commented on the visual image used or had not provided any suggestions for changes.

Third, we added Sense of Future as a life area to the IDUQOL with the following description: "e.g., hopefulness, aspirations, dreams, goals". The SMEs made several suggestions for life areas that could be added to the IDUQOL (i.e., food, pets, personal safety, sense of future, employment, pain). After considerable thought and given previous IDU focus group discussions about important life areas, we ultimately decided to only add Sense of Future as its own life area to the measure. However, we recognize that other researchers and practitioners may want to consider including these suggestions as additional life areas in their own work. Three points about our decision are worth noting: (a) following discussion, we decided that 'personal safety' was an implicit part of Neighborhood Safety, (b) we decided to incorporate 'pain' under our description of the Health life area, and (c) we resisted adding employment as its own life area because IDUs in a concurrent focus group study strongly opposed viewing employment as separate from, or more important than, other aspects of 'being useful in society'.

**Figure 2:**
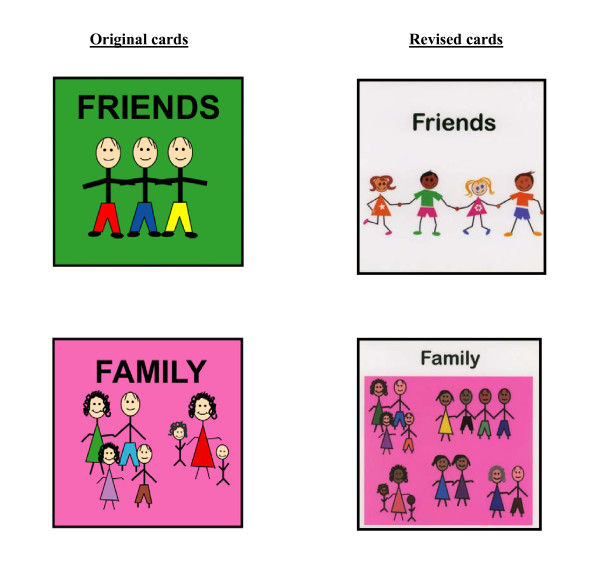
Original and revised cards to improve diversity of people.

**Figure 3:**
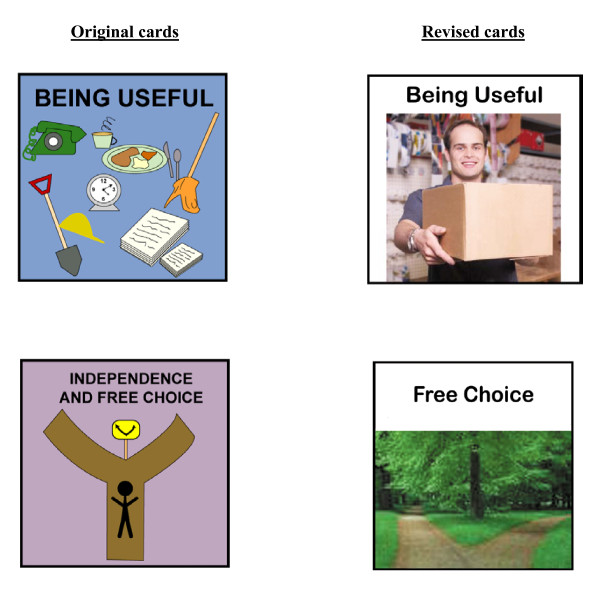
Original and revised cards to improve poor graphics on cards.

Fourth, we made revisions to the visual depictions for Friends, Family, Being Useful, and Free Choice. Based on SMEs' comments, we increased the diversity of people in the cards for Friends and Family (see Figure 2). For the Being Useful and Free Choice life areas, SMEs recommended using different images to make these concepts clearer to respondents. The old and new cards are shown in Figure 3.

Fifth, we were particularly struck by one SME's suggestion that the poker chips used in the response format for importance ratings might act as a trigger for IDUs with gambling issues and so we changed these to unmarked chips. We also incorporated suggestions made by SMEs for simplifying the instructions given to IDUs in the manual about how to use the chips to indicate the importance of the different life areas.

Sixth, we changed the Likert-type smiley face scale used to rate satisfaction from a 6-point scale to a 7-point scale that would permit a neutral response. This was based not only on SMEs' suggestions but also on our own concurrent experiences in administering the IDUQOL to IDUs [44,61]. We found that it was particularly appropriate to have a neutral option available when the respondent was rating satisfaction with a life area that had not been rated as particularly important.

**Figure 4:**
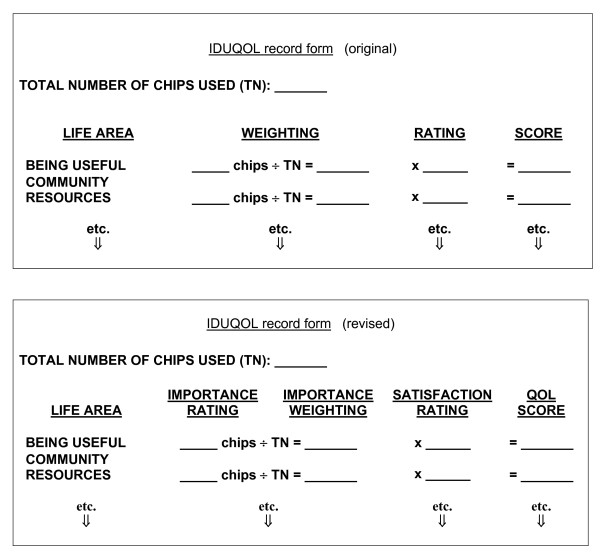
Original and revised IDUQOL record form headings.

Seventh, we revised the headings used in the IDUQOL record form so they would better match the terms used in the manual and would make obtaining the summed score easier (see Figure 4.)

Given the strong support provided by this study for the content validity of the IDUQOL and its manual, the revisions made to improve them further, and previous research supporting the validity of inferences made from the IDUQOL [44,61], the IDUQOL is a viable instrument for assessing broad-based QoL in IDUs and potentially in non-injection drug users [45]. The majority of published research in which QoL is examined with IDUs focuses on HRQOL. Future research is needed that examines the impact of drug use and various treatment options on QoL using broadly defined subjective QoL measures such as the IDUQOL. Future research on the IDUQOL needs to further examine its appropriateness and usefulness with non-injection drug users, its sensitivity to change, and its relationship with other broad-based QoL measures such as the Quality of Life Interview – Brief Version, which was originally developed for use with the mentally ill, or the Personal Wellbeing Index, which is the successor to the ComQol-A5 and was developed for use with the general population [62].

## Conclusion

The results from the present study provided strong support for the content validity of the elements of the IDUQOL measure and manual. Further revisions were made based on the CVI and AD_M _results as well as the open-ended feedback from the SMEs. These revisions included (a) revised names for three life areas (now Free Choice, Drugs & Alcohol, and Education & Training), (b) an expanded description for the Drugs & Alcohol, Education & Training, Family, and Sex life areas, (c) the addition of Sense of Future as a life area, (d) revisions to the visual depictions for Family, Friends, Being Useful and Free Choice, (e) the use of unmarked chips rather than poker chips in the response format for importance ratings, (f) the inclusion of a neutral point on the Likert-type scale of smiley faces for rating satisfaction, and (g) revisions to the headings used in the IDUQOL record form to make obtaining the summed score clearer. These revisions to the IDUQOL resulted in an instrument that is even easier for researchers, practitioners, and program evaluators to use as a way of assessing and tracking changes in QoL over time or as a result of interventions in IDUs.

## List of abbreviations

AD_M _Average Deviation Mean Index

CVI Content Validity Index

HRQOL health-related quality of life

IDUs injection drug users

IDUQOL injection drug user quality of life scale

QoL quality of life

SMEs subject matter experts

## Competing interests

The author(s) declare that they have no competing interests.

## Authors' contributions

AH obtained funding, designed the study, directed the statistical analyses, prepared the initial draft of the manuscript and conducted revisions. AP conceived of the study, obtained funding, coordinated data collection, and conducted revisions of the manuscript. Each author read and approved the final manuscript.
